# Regulating health and nutrition claims in the UK using a nutrient profile model: an explorative modelled health impact assessment

**DOI:** 10.1186/s12966-019-0778-5

**Published:** 2019-02-07

**Authors:** Asha Kaur, Peter Scarborough, Mike Rayner

**Affiliations:** Centre on Population Approaches for Non-Communicable Disease Prevention, Nuffield Department of Population Health, University of Oxford, Old Road Campus, Headington, Oxford, OX3 7LF UK

**Keywords:** Food labelling, Health claims, Nutrition claims, Nutrient profiling, NCD scenario modelling

## Abstract

**Background:**

Health-related claims (HRCs) are statements found on food packets that convey the nutritional quality of a food (nutrition claims) and/or its impact on a health outcome (health claims).

The EU stated that HRCs should be regulated such that they can only appear on foods that meet a specified nutrient profile (NP). A NP model has been proposed, but not agreed by the European Commission.

**Methods:**

To model the impact of HRCs on health impacts in the UK, we built a front-end model to a pre-established non-communicable-disease (NCD) scenario model, the Preventable Risk Integrated ModEl (PRIME) by combining data from a meta-analysis examining the impact of HRCs on dietary choices and a survey of pre-packaged foods examining the prevalence of HRCs and the nutritional quality of foods that carry them. These data are used to model the impact of regulating HRCs on the nutritional quality of the diet and PRIME is used to model the health outcomes associated with these changes. Two scenarios are modelled: regulating HRCs with a NP model (the FSANZ NPSC and a draft EU model) so that only foods that pass the model are eligible to carry HRCs, and reformulating HRC-carrying foods that fail the model.

**Results:**

Regulating the use of HRCs with a NP model (the FSANZ NPSC) would have unclear impacts on population health and could potentially lead to less healthy diets. This is because HRCs are currently more likely to be found on products with a better nutritional profile and restricting their use could shift consumers to less healthy diets. Two hundred fifty-eight additional deaths (95% Uncertainty Intervals [UI] -6509, 8706) were predicted if foods did not change in their nutrient composition. If all foods that currently carry HRCs were reformulated to meet the NP model criteria then there would be a positive impact of using the model: (4374 deaths averted (95%UI -2569, 14,009)). The largest contributor to the uncertainty is the underpowered estimates of nutritional quality of foods with and without claims.

**Conclusions:**

Regulating HRCs could result in negative health impacts, however the wide uncertainty intervals from this analysis demonstrate that a larger health impact assessment is necessary.

**Electronic supplementary material:**

The online version of this article (10.1186/s12966-019-0778-5) contains supplementary material, which is available to authorized users.

## Background

A poor diet is a major modifiable risk factor for NCDs [1]. Promoting healthier food choices is an important step to improving diet [2–4]. The European Commission (EC) has identified nutrition labelling as an important tool for consumers to make informed choices [5]. People who read the nutrition label tend to have more nutritionally favourable diets than people who do not read the nutrition label [6, 7]. However, the efficacy of nutrition labels to improve diet may be limited if it is only appealing to those who already have an interest in nutrition and/or health, or if the label can only be understood by those with advanced nutritional knowledge (sometimes referred to as the “nutrition elite”) [8]. Several systematic reviews have concluded that consumers would benefit from interpretative aids to help them understand nutritional information more easily [7, 9].

Health-related claims (HRCs) are verbal statements or visual images that refer to the nutritional content (nutrition claims) and/or health-promoting qualities of a food (health claims). A survey of foods available to purchase in the UK, Germany, the Netherlands, Spain and Slovenia found that 26% of foods (95% CI 24, 28%) carried at least one health-related claim (HRC) [10].

Evidence from experimental studies suggests that HRCs have a substantial impact on dietary choices [11]. HRCs may help to improve the diet if they help consumers identify and consume foods with a more favourable nutritional profile [12], but they may have detrimental effects on health if they encourage unhealthy dietary choices and/or excessive consumption of a product [13].

Within the EU, the use of HRCs is regulated by Regulation (EC) 1924/2006 [14]. In this regulation it states that a nutrient profile model should be used to prevent the overall nutritional quality of foods being masked by a health claim. Nutrient profiling can be defined as “… the science of classifying or ranking foods according to their nutritional composition for reasons related to preventing disease and promoting health” [15]. The algorithms used to make these classifications or ranks are called nutrient profile models.

A few countries already use a nutrient profile model to regulate HRCs, for example in Australia and New Zealand the Food Standards Australia New Zealand Nutrient Profiling Scoring Criterion (FSANZ NPSC) has been to regulate the use of health claims since 2016 [16]. A study of pre-packaged foods available to purchase in New Zealand found that 28% of foods categorised as ‘less healthy’ carried at least one HRC [17].

A recent analysis of the nutritional composition of foods in the EU that carry HRCs found that on average, these foods had a better nutritional profile than foods without claims: foods carrying HRCs had, on average, fewer calories, less saturated fat and, sodium, and significantly more fibre, per 100 g, than foods without HRCs [18]. If the HRCs on these foods provide a boost in sales [11] then they could be helping to improve the nutritional quality of the diet of the population and proposed regulation should be careful not to undermine this. Moreover, if restricting HRCs to foods that meet a nutrient profile model prompts foods that currently carry claims to reformulate in order to meet these criteria, then the nutritional gap between foods that carry and do not carry HRCs could widen, potentially improving the nutritional quality of the diet of the population even more.

The European Commission has yet to introduce a nutrient profile model for regulating HRCs and is currently reviewing Regulation 1926/2006. Part of this review involves assessing whether the regulation would be improved by the use of a nutrient profile model and estimating the cost of not having done so [19].

In June 2016 the UK voted to leave the EU and is now expected to formally leave the EU in March 2019. Whilst it is anticipated that most EU laws will continue as UK laws, the UK has the ability to amend or remove some EU laws. In this study we conduct a health impact assessment of the potential impact of using a nutrient profile model to underpin the regulation in the UK. It has been assumed that the regulation of HRCs with a nutrient profile model will have a positive impact, if any impact at all, through the restriction of less healthy foods. However, in a scenario where HRCs increase purchases of foods with a more favourable nutritional composition than foods without HRCs, it is possible that restricting the number of foods that can carry HRCs could have a negative impact.

### Aims

The research questions for this study are:

1. What would be the impact on NCD mortality rates in the UK if a nutrient profile model was used to underpin the HRC legislation so that only foods that pass a nutrient profile model were eligible to carry HRCs?
2. What would be the impact if foods that carry HRCs but fail the nutrient profile model were reformulated so that they meet the model criteria?

Two nutrient profile models were used to answer these questions: the nutrient profile model used to regulate HRCs in Australia and New Zealand (Food Standards Australia New Zealand’s Nutrient Profiling Scoring Criterion, FSANZ NPSC, [16]), and the latest version of the nutrient profile model proposed for use in Europe by the European Commission ([20], and Additional file 1: Supplementary Information).

## Methods

### Study design

For this study, we developed a model to estimate the impact of HRCs on diets (a HRC model) which was integrated with a pre-established NCD scenario model, the Preventable Risk Integrated ModEl (PRIME, [21]). The HRC model incorporates data from the EU funded project CLYMBOL (“Role of health-related CLaims and sYMBOLs in consumer behaviour” [22]).

**Fig. 1:**
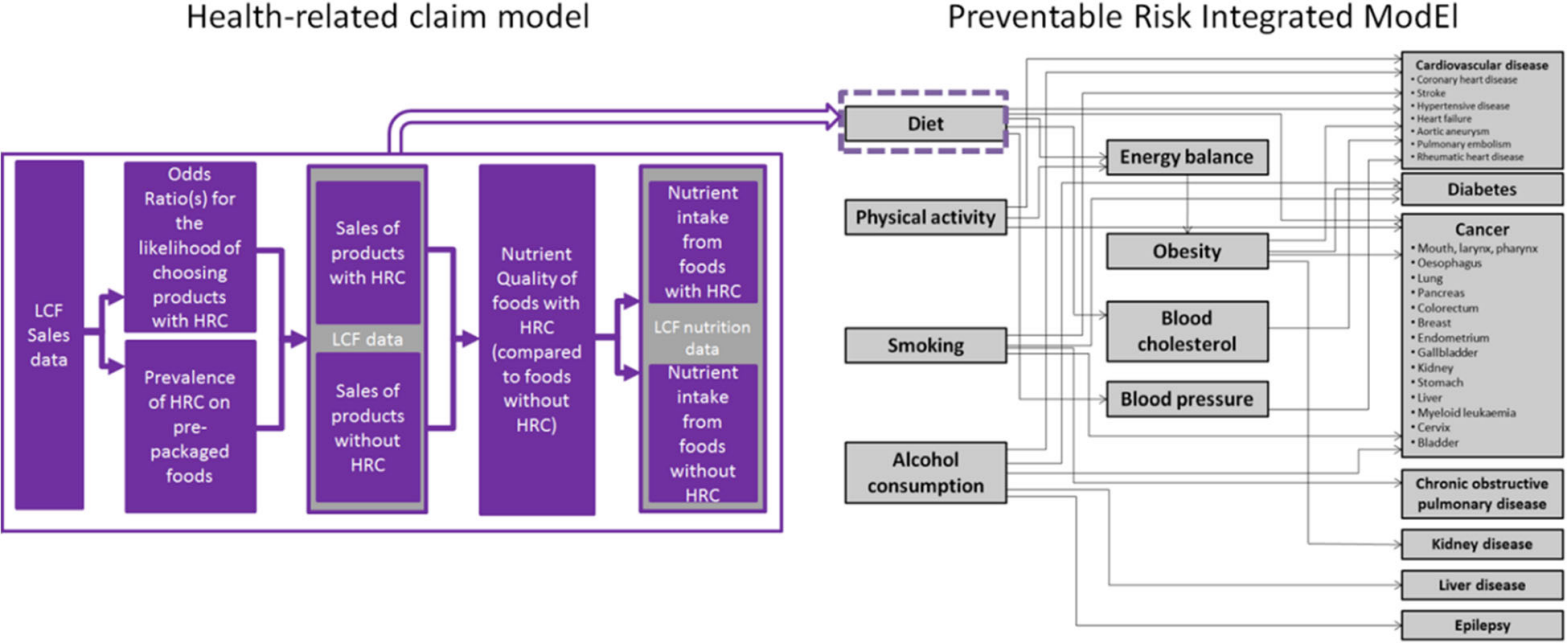
Prime Schematic and with Health-Related Claim Component. Abbreviations: HRC: health-related claim, LCF: Living Costs and Food Survey, PRIME: Preventable Risk Integrated ModEl (PRIME) developed by Scarborough et al. (2014)

PRIME estimates the number of deaths averted for 24 health outcomes. So far, this model has been used in 11 published studies to estimate the number of deaths averted or delayed under different conditions (e.g. [23]). The schematic diagram of PRIME, with the health-related claim component, is presented in Fig. 1.

PRIME links four behavioural risk factors (diet, physical activity, tobacco smoking, and alcohol consumption) to NCD mortality – in this analysis only the dietary mechanisms are included. The links between dietary risk factors and health outcomes can be direct or they can be mediated by three factors; body weight, blood cholesterol, and/or blood pressure.

PRIME estimates the number of deaths that would be delayed or averted following an intervention due to population changes in the prevalence of these behavioural risk factors. PRIME examines the current distribution of these risk factors and the levels of different diseases within a population and compares these to the distribution of the behavioural risk factors in the counterfactual scenario under investigation. PRIME then models the changes in the annual number of deaths based upon the differences between the baseline and counterfactual scenario, using population impact fractions (PIFs) [1, 24].

The links between the risk factors and the health outcomes were derived from a series of meta-analyses of studies examining the impact of changes in risk factors on disease outcomes. The relative risks used to characterise the links between the diet components and health outcomes are given in Scarborough et al. [21]. The components of diet included in PRIME are; fruit, vegetables, fibre, cholesterol, total fat, saturated fat, monounsaturated fat, poly-unsaturated fat, salt, and energy intake.

### Data sources

To configure PRIME to answer the research questions, the UK population and mortality data and UK population nutrient intake data are required. The population nutrient intake data are required to measure the current (baseline) distribution of nutrient intake within the population. These data were taken from the Living Costs and Food (LCF) survey (2015, [25]) and the National Diet and Nutrition Survey (NDNS, 2008–2012 [26]). The LCF survey is an annual survey of household expenditure conducted in the UK. The LCF measures the sales (in grams or ml) by food group, from this the nutrient intake is calculated by converting the grams of each food group purchased into nutrients from each gram purchased. The average nutrient intake is calculated using nutrient composition data from the UK Nutrient Databank [27].

To model changes in the distribution of dietary variables, the standard deviations (SD) of the mean nutrient intakes are required. This allows us to examine how the intake of nutrients is dispersed across the population. As these data are not provided in the LCF survey the SDs from the National Diet and Nutrition Survey (NDNS) were used instead [26]. The NDNS is a survey of food consumption in the UK. It collects detailed information on food consumption, nutrient intake, and nutritional status, which are collected from a representative sample of approximately 1000 people per year. The NDNS measures consumption of both unpackaged and pre-packaged foods. Whilst the NDNS data offers greater detail of food consumption then the LCF survey, the LCF is a more appropriate data source for this study’s analyses as it measures food purchases and the analyses presented in this study relate to pre-packaged foods available to purchase rather than food consumption.

The estimates for the population age and sex data were taken from the Annual Mid-Year Population Estimates of the UK (2013, [28]). The mortality data were collated from data for the registered deaths for England and Wales [29], Scotland [30], and Northern Ireland [31] .

The HRC data sources are summarised in Table 1.

**Table 1:** Description of health-related claim (HRC) data

Data	Methods	Findings
Impact of HRCs on dietary choices [11]	A systematic review of the effect of HRCs on pre-packaged foods, on dietary choices, including a meta-analyses of choice experiments which measured the likelihood of choosing a product when a HRC was present relative to when it was not.	Meta-analyses of 17 studies found that products carrying HRCs were 75% (OR 1.75, 95% CI 1.60, 1.91) more likely to be chosen than identical products without HRCs. Analyses by food group found that odds ratios differed by food group.
Prevalence of HRCs (the proportion of foods that carry at least one HRC) [10]	A randomly sampled selection of pre-packaged foods available to purchase in the UK, Germany, the Netherlands, Slovenia, and Spain. Approximately 400 products were sampled from each country. The nutritional information and HRC information was recorded from the food label.	Overall, 26% (95% CI 24, 28%) of foods carried a HRC, and the prevalence differed significantly between food groups.
Nutritional quality of foods carrying HRCs [18]	Using the same sample of foods described above [10], this study compared the mean levels, per 100 g of nutrients for foods carrying HRCs against foods that do not.	Compared to foods that did not carry any HRCs, foods with claims had, on average, significantly (*p* < 0.001) lower levels of; energy (− 43.7 kcal/100 g), protein (− 1.0 g/100 g), total sugars (− 3.2 g/100 g), saturated fat (− 2.9 g/100 g), and higher levels of fibre (+ 0.7 g/100 g p < 0.001), and lower levels of sodium (− 354 mg/100 g, *p* < 0.006)
The impact of using nutrient profile models to regulate the use of HRCs [18]	Using the same data above [10, 18] this study assessed the impact of restricting health claims with a nutrient profile model. This was assessed as difference in the mean level of nutrients of foods that carry health claims and pass the FSANZ NPSC and foods that do not. For our study we looked at the mean level of nutrients for foods that carry HRCs and pass the FSANZ NPSC, and the EU model, and foods that do not.	Foods that carry health claims had, on average per 100 g, significantly (*p* < 0.01), fewer calories (− 29 g), less protein (− 1 g), total sugars (− 3 g), significantly (p < 0.001), less saturated fat (− 2 g) and sodium (− 842 mg) and more fibre (+ 0.8 g). Using the FSANZ NPSC to restrict health claims resulted in greater differences but this varied by nutrient. Foods that carried health claims and passed FSANZ NPSC had significantly (p < 0.001) fewer calories (56 kcal), less protein (− 1.6 g), total sugars (− 7.3 g), saturated fat (− 2.9 g), sodium (− 977.6 mg), and more fibre (+ 1 g), relative to foods without claims.

### Baseline scenario

Earlier studies have identified food group as a potential confounder to the relationship between the nutritional quality of foods and whether or not they carry HRCs as the prevalence of HRCs differs by food group and the nutritional composition of foods differs between food groups [18]. Therefore, in this study each of the HRCs parameters is stratified by the food groups used in the Eatwell Guide [32]. The Eatwell Guide forms the basis to the UK Government’s food based dietary guidance. The Eatwell Guide has the following groups:

- potatoes, bread, rice, pasta and other starchy carbohydrates,
- foods high in fat, salt, and sugar,
- oils and spreads,
- dairy and alternatives,
- beans, pulses, fish, eggs, meat and other proteins,
- fruit and vegetables.

In this study two additional groups were created to categorise foods that were not represented in the Eatwell Guide:

- ‘composite foods’: foods that could fit into more than one category such as pizzas or ready meals.
- ‘miscellaneous foods’: foods that do not fit one of the above categories e.g. tea, coffee, water, and seasonings,

The baseline scenario reflects the current nutrient intake in the UK and the current UK mortality rates/burden of disease. This scenario assumes that the current nutrient intake and NCD mortality rates reflect a situation where, (depending on the food group), 22–67% of foods carry at least one HRCs and that these foods have the nutritional qualities as estimated in Kaur et al. [18].

**Fig. 2:**
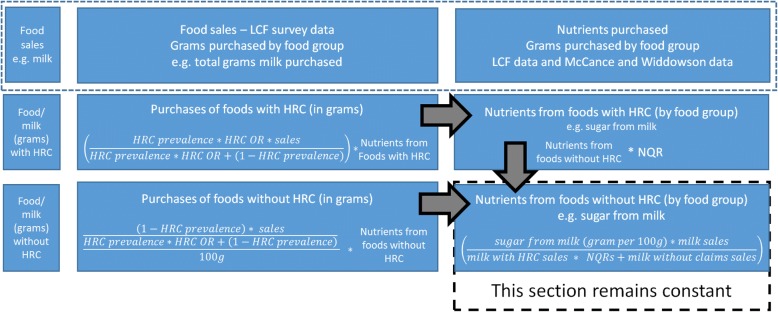
Equations Used to Disaggregate The Living Costs and Food (LCF) Survey Data Abbreviations: HRC: health-related claim, LCF: Living Costs and Food Survey, NQR: nutrient quality ratio, OR: odds ratio

The LCF data on the sales of products is divided into sales of products with HRCs and sales of products without HRCs. This is achieved through solving mathematical equations (Fig. 2) combining the LCF sales data with Odds Ratios for the likelihood of choosing a product with a HRC [11] and HRC prevalence estimates [10], so that the sum of the sales of products with HRCs and the sales of products without HRCs is equal to the total sales of products (as estimated by the LCF).

The division of the nutritional intake into nutrients from foods with HRCs and nutrients without HRCs is done with a similar method. Using data, from Kaur et al. [18], the mean level of nutrients, per 100 g, for foods with HRCs are divided by the mean level of nutrients for foods without claims to create what we refer to as a ‘Nutrient Quality Ratio’ (NQR). Mathematical equations are then solved that combine the NQRs with the sales of foods carrying and not carrying HRCs, so that the nutrients from foods with and without HRCs can be estimated, and such that the total nutritional quality of the baseline diet matches that shown in the LCF dataset. The equations and steps taken to disaggregate the LCF survey data is presented in Fig. 2. This allocation of sales and nutrients between foods that carry HRCs and those that do not is required to model the remaining scenarios.

### Descriptions of the models

Two scenarios are modelled, ‘HRCs restricted’ (Models 1a, 2a) and ‘reformulating foods to carry HRCs’ (Models 1b, 2b). Under the ‘HRC restricted’ scenarios HRCs are restricted using a nutrient profile model so that only foods that pass the FSANZ NPSC (Model 1a) and the nutrient profile model proposed by the EU (Model 2a) may carry HRCs. In these models foods that currently carry HRCs but fail the nutrient profile model(s) are considered not to carry HRCs any longer and hence no longer receive a boost in sales. The nutritional quality of the foods that lose HRCs are assumed to change to the average nutritional quality of foods that do not carry HRCs.

Under the ‘reformulating foods to carry HRCs’ models the use of HRCs is restricted with a nutrient profile model, but the HRC prevalence is maintained at current levels. Here, foods that currently carry HRCs but would have them removed as they do not satisfy the nutrient profile are assumed to reformulate in order to pass the nutrient profile model (FSANZ NPSC: Model 1b, EU Model: Model 2b) and retain the HRC. Detailed descriptions of the models are available in the Additional file 1: Supplementary information.

In each model the HRC prevalence and the NQRs are recalculated to produce specific results for each nutrient profile model being tested. The calculated nutrient composition (per 100 g) of foods not carrying HRCs remains the same as in the baseline scenario, and is multiplied by the new NQRs to achieve the nutrient composition (per 100 g) of foods carrying HRCs. The parameters for each model are outlined in Table 2.

**Table 2:** Parameters used to model the impact of health-related claims (hrcs) on uk mortality from non-communicable diseases (95% confidence intervals)

Food group	Impact of HRCs on dietary choices (ORs) [11]	HRCs prevalence		
		‘HRCs restricted’ models		‘HRCs restricted & reformulated’ models
		1a FSANZ NPSC	2a EU model	1b FSANZ NPSC 2b EU model (Baseline HRC prevalence [10]
Potatoes, bread, rice, pasta or other starchy carbohydrates	1.17 (1.60–1.91)	19% (14–25%)	19% (14–25%)	29% (23–36%)
Composite foods	1.06 (0.91–1.24)	12% (8–17%)	14% (10–20%)	16% (12–22%)
Foods and drinks high in fat and/or sugar	1.35 (1.09–1.66)	11% (9–13%)	11% (9–14%)	23% (20–26%)
Fruit and vegetables	1.92 (1.56–2.35)	31% (25–39%)	30% (23–37%)	33% (26–41%)
Beans, pulses, fish, eggs, meat and other protein	2.42 (1.87–3.12)	9% (6–13%)	12% (8–16%)	18% (14–22%)
Dairy or dairy alternatives	1.25 (1.22–1.27)	37% (30–45%)	40% (32–47%)	46% (39–54%)
Miscellaneous	1	18% (14–23%)	25% (21–31%)	31% (26–37%)

### Uncertainty analyses

Monte Carlo analyses were used to calculate the 95% Uncertainty Intervals (UI) around the point estimates. The Monte Carlo analyses were set at 10000 iterations and incorporated the uncertainty around the following variables:

- the prevalence of HRCs,
- the mean nutrients per 100 g for foods that do and do not carry HRCs,
- the Odds Ratios (ORs) for the impact of HRCs on dietary choices,
- the Relative Risks (RRs) for the epidemiological parameters used in PRIME.

## Results

### The impact of health-related claims on the average population nutrient intake

The average daily nutrient intake from foods with and without HRCs are provided in the Additional file 1: Supplementary Information. In the baseline scenario, foods carrying HRCs made-up 37% of the total purchases, and contributed 29% (559 kcal/person/d) of the total number of kcals purchased (1907 kcal/person/d). Relative to the baseline scenario, in the ‘*HRCs restricted*’ models there is a 10% point reduction in the proportion of purchases of foods that carry HRCs. Under Model 2a there is a 39% reduction (95% UI 20, 51%), under Model 1a there is a 46% reduction (95% UI 28, 59%), in energy from foods carrying HRCs.

The nutrient intake stratified by Eatwell Guide food group is presented in the Additional file 1: Supplementary Information. The difference in nutrient intake (relative to the baseline scenario) is presented in Table 3.

**Table 3:** Difference in nutrient intake (per day) under each health-related claim (HRC) scenario relative to baseline scenario (95% uncertainty intervals)

		‘HRCs restricted models’		‘HRCs restricted and reformulated models’	
	Baseline nutrient intake [25]	FSANZ NPSC 1a	EU model 2a	FSANZ NPSC 1b	EU model 2b
Energy (kcal)	1906.8	−18.3 (−54.2, 18.6)	−7.9 (− 45.4, 31.3)	−90.1 (− 142.8,35.8)	−60.2 (− 112.1, − 6.6)
Protein (g)	66.1	+ 0.3 (− 1.4, 2.2)	+ 0.7 (− 1.1, 2.6)	− 1.0 (− 3.3, 1.5)	−0.2 (− 2.3, 2.2)
Total fat (g)	79.7	+ 1.6 (− 1.2, 4.5)	+ 1.4 (− 1.6, 4.5)	−2.5 (− 7.7, 2.7)	−1.8 (− 6.7, 3.2)
Saturated fat (g)	31.0	+ 1.5 (0.4, 2.5)	+ 1.3 (0.2, 2.3)	−2.2 (− 3.6, − 0.9)	−1.6 (− 3.0, − 0.3)
Carbohydrates (g)	233.8	−6.0 (− 11.7, 1.2)	−5.3 (− 10.9, 2.0)	−11.1 (− 18.8, − 2.2)	−8.8 (− 16.5, 0.5)
Total sugars (g)	105.9	−2.2 (− 8.0, 5.9)	− 3.3 (− 8.9, 4.6)	−10.7 (− 17.3, − 1.7)	−10.9 (− 17.6, − 1.8)
Fibre (g)	12.6	−0.2 (− 1.3, 1.6)	− 0.5 (− 1.5, 1.1)	+ 0.6 (− 0.9, 2.9)	+ 0.2 (− 1.1, 2.3)
Sodium (g)	2.3	0.0 (− 0.1, 0.1)	−0.1 (− 0.2, 0.0)	−0.2 (− 0.3, − 0.1)	−0.2 (− 0.4, − 0.1)
Fruit (g)	151.2	+ 3.7 (− 30.4, 58.8)	−3.6 (− 35.5, 48.4)	+ 5.5 (− 30.1, 64.7)	−0.6 (− 35.5, 55.9)
Vegetables (g)	131.1	+ 4.0 (− 13.8, 23.0)	+ 8.2 (− 8.9, 27.0)	+ 3.3 (− 15.2, 22.9)	+ 7.1 (− 12.0, 28.0)

Under Model 1a, there were minor changes (from baseline) in protein (+ 0.3 g/d, 95% UI -1.4, 2.2) fibre (− 0.2 g/d 95% UI -1.3, 1.6), and fat (+ 1.6 g/d, 95% UI -1.2, 4.5). Greater changes were seen for total sugars (− 2.2 g/d, 95% UI -8.0, 5.9), carbohydrate (− 6 g/d, 95% UI -11.7, 1.2), fruit (+ 3.7 g/d, 95% UI -30.4, 58.8) and vegetables (4.0 g/d, 95% UI -13.8, 23.0).

Under Model 2a there were changes of less than 1 g/d in the levels of fibre (− 0.5 g/d, 95% UI -1.5, 1.1), protein (+ 0.7 g/d, 95% UI -1.1, 2.6), and sodium (− 0.1 g/d, 95% UI -0.2, 0.0). Saturated fat increased by 1.3 g/d (95% UI 0.2, 2.3). Larger changes were seen for total sugars (− 3.3 g/d, 95% UI -8.9, 4.6), carbohydrates (− 5.3 g/d, 95% UI -10.9, 2.0), fruit (− 3.6 g/d, 95% UI -35.5, 48.4), and vegetables (+ 8.2 g/d (95% UI -8.8, 27.0).

Under the ‘*reformulating foods that carry HRCs*’ models (Models 1b, 2b) there were reductions in energy intake of 90 kcal/d (Model 1b, 95% UI -142.8, − 35.8) and 60 kcal/d (Model 2b, 95% UI -112.1, − 6.6). There were reductions in the total fat content under both models. There were also large reductions in the total sugar intake: 10.7 g/d (95% UI -17.3, − 1.8) for Model 1b, and 10.9 g/day (95% UI -17.6, − 1.8) for Model 2b.

Under Model 1b fruit intake and vegetable intake increased by 5.5 g/d (95% UI -30.1, 64.7) and 3.3 g/d (95% UI -15.2, 22.9) respectively. Under Model 2b there was a small reduction in in fruit (− 0.6 g/d, 95% UI -35.5, 55.9) but a large increase in vegetables (7.1 g/d, 95% UI –12.0, 28.0).

### Health outcomes

The modelled impact of HRCs on UK NCD mortality rates are presented in Table 4. This table presents the number of deaths delayed or averted, with negative numbers indicating that the number of deaths would be increased from the baseline scenario.

**Table 4:** The Impact Of Health-Related Claims (HRCs), In Different Regulatory Scenarios, On Uk Mortality From Non-Communicable Diseases (95% Uncertainty Intervals)

	‘HRCs restricted’ models		‘HRCs restricted & reformulated’ models	
Deaths averted or delayed:	1a – FSANZ NPSC	2a - EU model	1b – FSANZ NPSC	2b - EU model
Total	−258 (−6509, 8706)	− 782 (− 6800, 7705)	4374 (− 2569, 14,009)	3151 (− 3783, 12,296)
Male	− 277 (− 3582, 4460)	− 481 (− 3662, 3970)	2363 (− 1347, 7455)	1743 (− 1960, 6583)
Female	19 (− 2946, 4286)	− 301 (− 3134, 3712)	2011 (− 1259, 6533)	1408 (− 1843, 5795)
Deaths averted or delayed by cause:				
Cardiovascular disease	−347 (− 5565, 6946)	− 573 (− 5539, 6308)	4078 (− 1708, 12,085)	3136 (− 2793, 10,710)
Cancer	89 (− 1236, 2030)	− 209 (− 1511, 1681)	295 (− 1067, 2287)	15 (− 1388, 2039)
Deaths averted or delayed by behavioural risk factor:				
Fruit and vegetables	995 (− 4737, 9177)	290 (− 5179, 8246)	1198 (− 4729, 9478)	656 (− 5394, 9068)
Fibre	− 366 (− 1989, 2651)	−750 (− 2275, 1815)	982 (− 1295, 5045)	319 (− 1723, 3793)
Fats	− 1025 (− 1768,-349)	− 787 (− 1541, − 71)	182 (−947, 1229)	180 (− 922, 1197)
Salt	129 (− 579, 1020)	−455 (− 285, 1486)	2052 (716, 3776)	2016 (651, 3781)
Deaths averted or delayed when changes in energy intake are taken into account				
Total	996 (− 5632, 9519)	−228 (− 6803, 8205)	9450 (2468, 18,851)	6795 (− 203, 15,755)

Using a nutrient profile model to restrict HRCs would lead to a < 1% increase in deaths compared to the baseline scenario. Regardless of which NP model is used for the regulation, our models estimated that regulating HRCs would lead to a small increase in deaths, but with a large degree of uncertainty in the results. If the FSANZ NPSC model was used then there would be an additional 258 deaths (95% UI -6509, 8706) and a greater number of additional deaths are estimated with the EU model (− 782, 95% UI -6800, 7705).

If a nutrient profile model is used to regulate HRCs but the prevalence of HRCs is maintained at current levels – reflecting a situation where manufacturers reformulate foods that do not currently meet the model criteria – our models predict a < 2% reduction in deaths from the baseline. A positive health impact is observed for both nutrient profile models, but still with wide uncertainty intervals. Under the FSANZ NPSC model there would be 4374 (95% UI -2569, 14,009) deaths averted and under the EU model there would be 3151 (95% UI -3783, 12,296).

### Uncertainty analyses

**Fig. 3:**
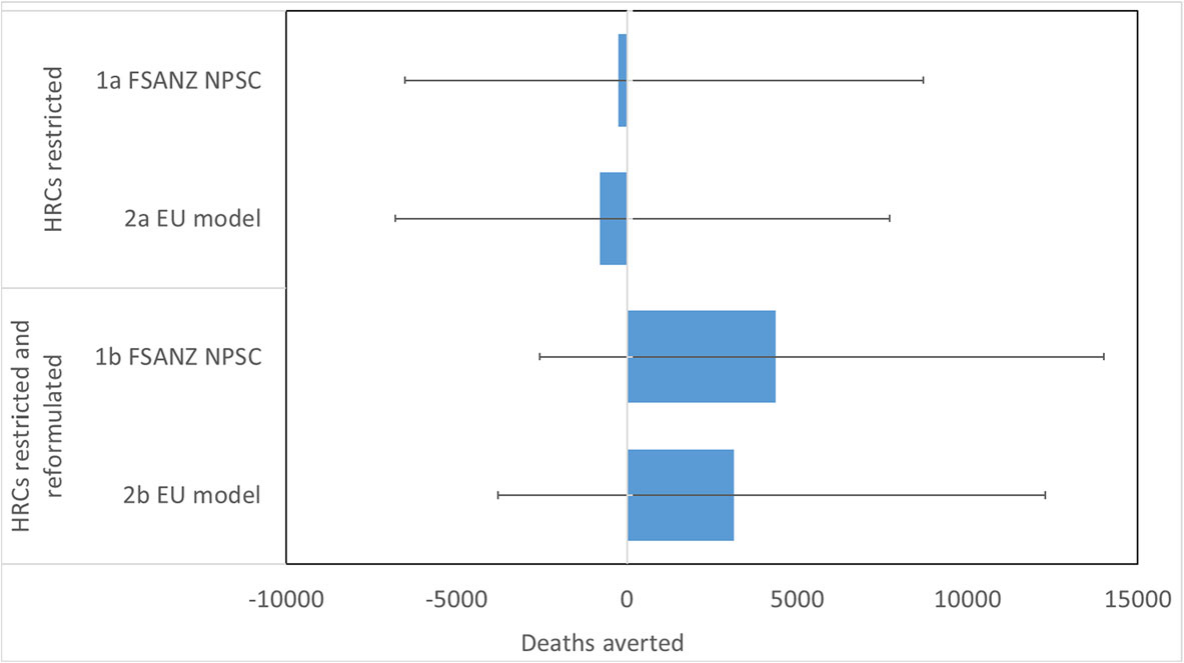
The Impact Of Health-Related Claims, In Different Regulatory Scenarios, On UK Mortality From Non-Communicable Diseases. Abbreviations: HRCs: health-related claims, FSANZ NPSC: Food Standards Australia New Zealand Nutrient Profiling Score Criterion. Note: The impact of HRCS in different regulatory scenarios, as predicted by the PRIME model (Scarborough et al., 2014). Here a negative number indicates that the number of deaths would be increased from the baseline scenario

**Fig. 4:**
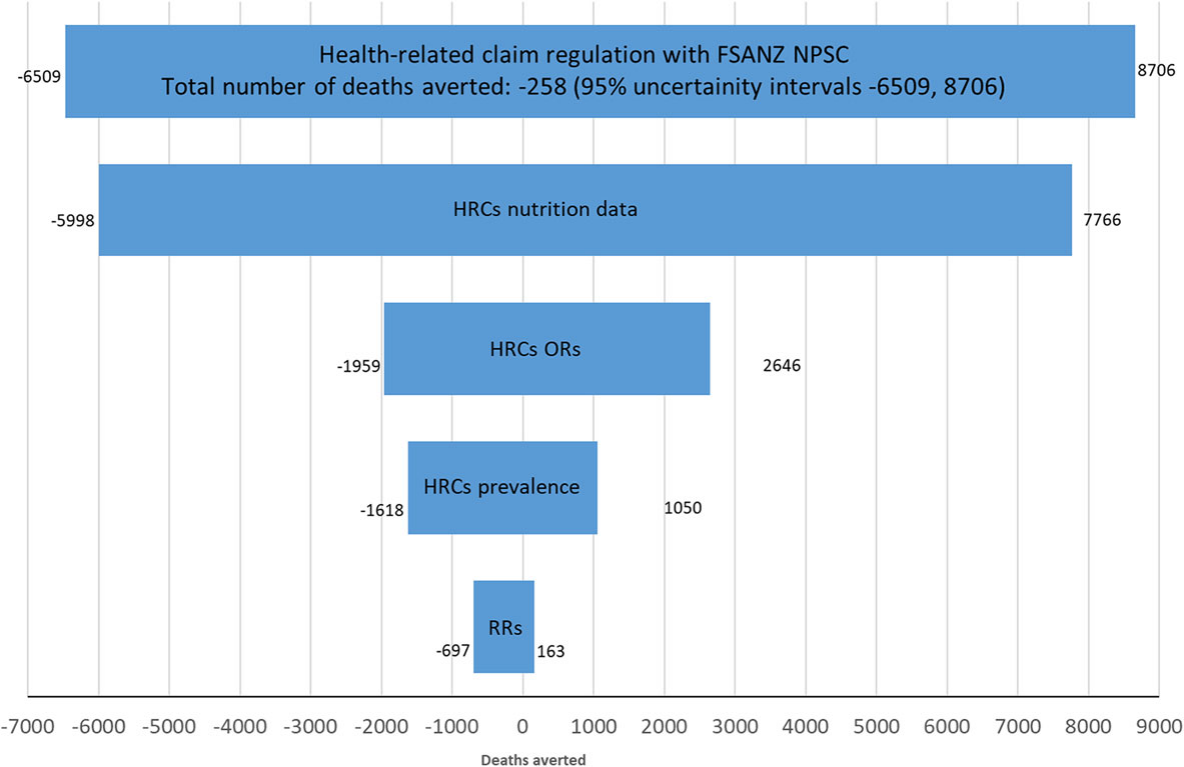
Tornado plot showing the variance around the parameters under Model 1a (regulating HRCS with FSANZ NPSC). Abbreviations: HRCs: health-related claims, FSANZ NPSC: Food Standards Australia New Zealand Nutrient Profiling Score Criterion, OR: odds ratio, RRs: relative risks associated with the nutrient intake for the dietary risk factors and the health outcomes used by PRIME. Note: Model 1a refers to the scenario where the use of HRCs are restricted so that only foods that pass the FSANZ NPSC may carry HRCs

The uncertainty intervals observed in this study are large and in most instances they cross zero. A tornado plot analysis of Model 1a (Fig. 3) was conducted to assess which parameters contributed the most uncertainty to the model. Here, ‘RRs’ refers to the relative risks associated with the nutrient intake for the dietary risk factors and the health outcomes used by PRIME. Almost all of the uncertainty is due to the data on which the nutrient quality ratios are devised i.e. the variance in the mean level of nutrients of foods that do and do not carry HRCs as recorded from the food labels (Fig. 4).

## Discussion

### Summary of main findings

When considering the main findings of this study, it is important to recognise that the wide uncertainty intervals demonstrate that this dataset is not very suitable for making definitive statements about the health outcomes from different regulatory scenarios. However the scenarios that were modelled here have not been analysed before and the methods that have been employed are innovative. The dataset used was the largest representative dataset of foods where HRCs are identified that is available. Furthermore the large size of the uncertainty intervals could not have been predicted in advance – it is not possible to conduct power calculations for modelling studies of this kind.

The modelling study does reveal an unexpected prospect: that regulating health and nutrition claims with a nutrient profile model *could* (but we are far from certain that it *would*) result in less healthy diets. This possibility is mainly dependent upon the earlier finding that foods with HRCs have greater nutritional quality on average than foods without HRCS. This finding, combined with the increased likelihood of purchases associated with HRCs, means that restriction of HRCs using nutrient profile models can lead to unexpected outcomes. This is why a more appropriately powered health impact assessment is essential if a nutrient profile model for regulating HRCs is to be introduced in Europe.

The finding that most of the uncertainty in the model was around the food composition data highlights the need for larger surveys of HRCs. Techniques to develop large food databases are already being employed, for example data mining/scraping nutritional and other information from retailers’ and manufacturers’ websites, and crowd-sourcing food label data through the use of mobile phone apps (e.g. [33]). However, automated methods for the detection and categorisation (e.g. through machine learning) of HRCs on food labels that can be applied to large datasets still need to be developed and may require large food training datasets.

The analyses here show that manufacturers’ responses to HRCs (i.e. whether or not they reformulate foods to maintain the prevalence of HRCs) is an essential factor that will determine the success of regulations restricting the use of HRCs. However, there is no clear evidence how manufacturers would respond. Currently there are a small number of studies examining the impact of voluntary health-related logos on reformulation (e.g. [34–36]) but there are no studies that have investigated the impact of HRC regulation on reformulation.

### Previous research

There is little previous research that is relevant to the conclusions of this study. In the only relevant study we could identify Vyth (2012, [37]) modelled the effect of a particular health-logo – a particular type of HRC - on blood cholesterol levels in the Dutch population. In that study foods that were not eligible to carry the logo (the Choices logo) were replaced with foods that did meet the nutritional requirements of the program. The effect on blood cholesterol levels was estimated by combining the data on the nutritional differences between the current diet and the modelled diet and meta-analyses of studies concerning the effect of diet on cholesterol levels. From this, Vyth concluded that only moderate changes in blood cholesterol levels would be predicted. However, this study only looked at one health outcome and one type of HRC whereas this study looked at HRCs and their effect on mortality from numerous NCDs.

There is a lack of understanding of how consumers respond to HRCs in the real-world, for example consumers, when faced with HRCs, may engage in compensatory behaviours such as consuming more foods with the claim (rather than substituting a food with claim for one without). Similarly with manufacturers – whilst previous studies suggest that manufacturers are willing to reformulate foods [38], and also willing to develop new products that meet health logo requirements [34, 36] this is not a certainty, and manufactures could reformulate foods to make them less healthy. Further research is required on manufacturers’ responses to such legislation. A comprehensive study was conducted examining manufacturers’ responses to the Choices logo by Vyth et al. (2010, [61]). The study found that around half of the products carrying the Choices logo were formulated or reformulated in order to comply with the model. This study involved 47 food manufacturers who participated in the scheme and they may not have been be representative of all manufacturers.

### Strengths and limitations of the study

One of the assumptions of the ‘HRCs restricted’ models (models 1a, 2a) is that foods that (previously) carried HRCs but fail the respective nutrient profile model, change to having the nutritional quality of foods that do not carry HRCs. This may mean that we have underestimated the nutritional quality of foods that do not carry HRCs, as the nutritional quality of this group may improve with the new addition of foods that previously carried HRCs (as these foods that previously carried HRCs are more likely to have a more favourable nutritional composition than foods that do not carry HRCs).

The application of the nutrient profile models does not necessarily reflect how the models would be applied in reality. For example, the FSANZ NPSC model was applied uniformly to all foods and all types of HRCs, but in Australia and New Zealand, the model is not applied to foods carrying nutrition claims, only to foods carrying health claims, and there are additional requisites for many types of HRC. For example, to carry a health claim that refers to the impact of high fruit and vegetable intake and the reduced risk of developing coronary heart disease, the product carrying the claim must not be a fruit juice and must contain at least 90% fruit and/or vegetable (Schedule 2, [16]). To incorporate these types of requisites would have required further data collection and more detailed HRC categorisation. Therefore, it is likely that our HRCs model is more or less restrictive in reality which may impact the results presented here.

It is possible that the odds ratios for the likelihood of choosing a food when a HRC was present (relative to when it is not) used to parameterise the model over-estimate the effect of HRCs. Natural experiments examining HRCs tend to find smaller effects. For example, in a natural experiment with real food sales, Kiesel (2013, [39]) examined the effect of nutrition claims on snack product sales and found that on average there was a 16% increase in sales for products carrying claims. An equivalent estimate from a meta-analysis of all studies (both natural experiments and experiments in artificial settings) suggests a 43% increase [11]. We investigated this with a sensitivity analyses and found that by assuming that HRCs increase purchasing by 16% (across all food categories) in the baseline scenario and in the ‘HRCs restricted scenario’, our model predicted that there would be an additional 571 deaths if HRCs were restricted using the FSANZ NPSC. This is more than double than what is predicted under model 2a (− 258 deaths, 95% UI -6509, 8706) where we assume that HRCs increase purchasing using the ORs presented in Table 2.

The odds ratios used assume that HRCs affect the population universally. Age and/or gender-specific odds ratios were not available due to a lack of this information in the literature. Previous systematic reviews suggest that the impact of HRCs is greater on younger women in higher socio-economic groups [9, 40, 41] but none of these studies provide a quantified effect size.

This study assumes that the overall food consumption (in g/day) does not change and any food substitutions are made within food category. There is a lack of data on how HRCs may impact on total food consumption or how HRCs may impact on consumption of other food categories. It is possible that consumers may engage in compensatory behaviour, or may shift to other food categories.

The estimates for the prevalence of HRCs were derived from a survey of pre-packaged foods available to purchase in the EU. Sales of products were not included in the sampling frame, this could mean that the prevalence estimates under-estimate the population’s exposure to HRCs [42].

This modelling study is subject to the limitations of the data which populate the model. For example, the LCF survey [25] and the NDNS [26] data are used to estimate current nutrient intake, however, under-reporting of nutrient intake is a known limitation of most dietary surveys [43].

This study did not examine the pricing of foods with HRCs. HRCs could potentially increase social inequalities as previous research suggests that higher socioeconomic groups have stronger preferences for HRCs and consumers may be willing to pay more for foods that carry HRCs [40, 41]. Whilst this out of the scope of this study, future research should examine whether there may be any socio-economic consequences of employing and regulating HRCs.

## Conclusions

The results of this study suggest that restricting the use of HRCs with a nutrient profile model could result in either negative or positive health impacts. Manufacturers’ responses to the introduction of the nutrient profile model will effect that impact and determine the success of the restrictions. Moreover the wide uncertainty intervals from this analysis demonstrate that a larger health impact assessment is necessary.

## Additional file

Additional file 1: Supplementary information including further background information, description of model scenarios, and results. (PDF 855 kb)

## Abbreviations

/day: per day
CI: Confidence Intervals
EC: European Commission
EU: European Union
FSANZ NPSC: Food Standards Australia New Zealand Nutrient Profiling Scoring Criterion
FSANZ: Food Standards Australia New Zealand
g: grams
HRC: health-related claim
HRCs: health-related claims
kcal: kilocalories
LCF: Living Costs and Food survey
NCD: non-communicable-disease
NDNS: National Diet and Nutrition Survey
NQR: nutrient quality ratios
OR: odds ratio
PIF: population impact fractions
PRIME: Preventable Risk Integrated ModEl
RR: relative risk
UC: Uncertainty Intervals
US FDA: United States Food and Drug Administration
