# Supplementary material for: Regulating health and nutrition claims in the UK using a nutrient profile model: an explorative modelled health impact assessment
Source: Int J Behav Nutr Phys Act. 2019 Feb 7;16:18. doi: 10.1186/s12966-019-0778-5 (PMC6367824; doi:10.1186/s12966-019-0778-5)
Supplement: Supplementary file 1 — Supplementary information including further background information, description of model scenarios, and results. (PDF 855 kb) [file 12966_2019_778_MOESM1_ESM.pdf]

Supplementary information for 'Regulating health and nutrition claims in the UK using a nutrient profile model: an explorative modelled health impact assessment'

Dr Asha Kaur, Dr Peter Scarborough, Professor Mike Rayner.
Centre on Population Approaches for Non-Communicable Disease Prevention
Nuffield Department of Population Health, University of Oxford

BACKGROUND 1
PRIME 1
METHODS 2
Description of scenarios 2
Figure 1. Baseline scenario 2
Figure 2. Health-related claims restricted (Models 1a, 2a) 3
Figure 3. Health-related claims restricted and reformulated (Models 1b, 2b) 3
Draft EU nutrient profile model for the regulation of health and nutrition claims 4
RESULTS 5
Table 1. Nutrient intake (per day) under each health-related claim (HRC) scenario 5
Table 2. Nutrient intake (by food group) under each health-related claim (HRC) scenario, purchase averages g/ml per person, per week (LCF 2014) 6

BACKGROUND

Non-communicable disease (NCD) modelling involves examining different behaviours/risk factors ('inputs'), such as dietary behaviour, and the associated risk of developing and/or dying from a disease ('outcomes'). NCD modelling can be used for a variety of purposes including to inform decision-making (e.g. modelling cost effectiveness of different interventions/polices such as taxes on less healthy foods and/or subsides on healthier foods[1]), and estimating future disease incidence and mortality trends[2].

PRIME

In this study, we build upon a pre-established NCD scenario model, the Preventable Risk Integrated ModEl (PRIME[3]) to model the impact of nutrient intakes associated with different food labelling scenarios on UK mortality rates.

---

[1] Cobiac, L.J., et al., *Taxes and Subsidies for Improving Diet and Population Health in Australia: A Cost-Effectiveness Modelling Study.* PLoS Med, 2017. **14**(2): p. e1002232.
[2] Webber, L., et al., The Brighton declaration: the value of non-communicable disease modelling in population health sciences. Eur J Epidemiol, 2014. 29(12): p. 867-70.
[3] Scarborough, P., et al., *The Preventable Risk Integrated ModEl and Its Use to Estimate the Health Impact of Public Health Policy Scenarios.* Scientifica (Cairo), 2014. **2014**: p. 748750.

PRIME estimates the number of deaths averted for 24 health outcomes, these are grouped into the following categories, cardiovascular disease (CVD), diabetes, cancer, chronic obstructive pulmonary disease (COPD), kidney disease, and liver disease. This model has been used in 11 published studies to estimate the number of deaths averted or delayed under different conditions. For example, for the UK it was estimated that 33000 deaths per year would be avoided if UK dietary recommendations were met[4].

METHODS

This section provides additional information on how we modelled each scenario.

Description of scenarios

Figure 1. Baseline scenario

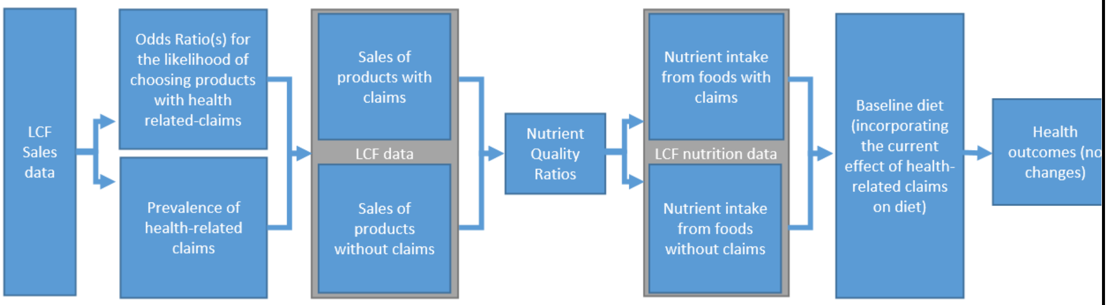

As health-related claims (HRCs) are already present on foods it is assumed that any impact that they may have upon diet - and subsequently, health outcomes - are already in effect.

[4] Scarborough, P., et al., *Modelling the impact of a healthy diet on cardiovascular disease and cancer mortality.* Journal of Epidemiology and Community Health, 2010. **66**: p. 420-426.

Figure 2. Health-related claims restricted (Models 1a, 2a)

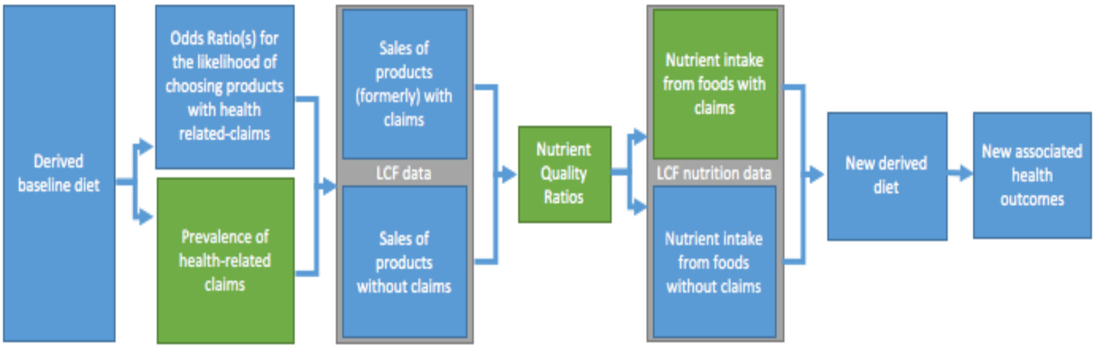

In Models 1a and 2a the use of HRCs is restricted using a nutrient profile model so that only foods that pass the FSANZ NPSC (Model 1a) or the nutrient profile model proposed by the EU (Model 2a) may carry health-related claims. In these Models foods that currently carry HRCs but fail the nutrient profile model(s) are considered not to carry a HRC any longer. For example, if 26% of foods carry HRCs, but 38% of these foods fail the FSANZ NPSC, the expected prevalence of HRCs under the FSANZ NPSC would be reduced to 16%.

Figure 3. Health-related claims restricted and reformulated (Models 1b, 2b)

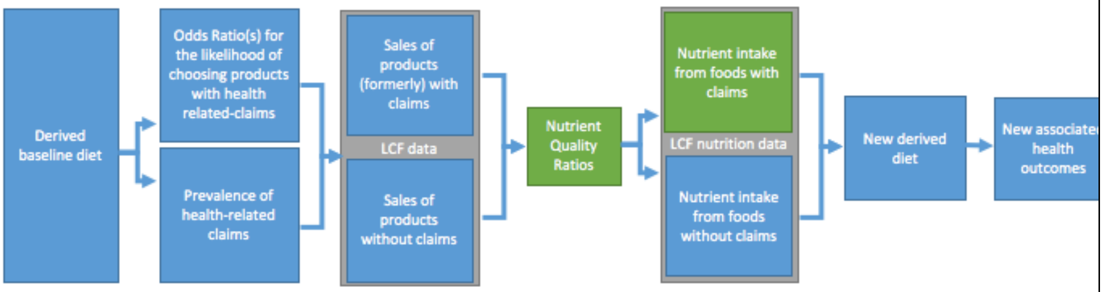

In these scenarios the use of HRCs is restricted with a nutrient profile model, but the HRC prevalence is maintained at current levels. Thus, these models suggest that manufacturers of foods that currently carry claims but fail to meet a nutrient profile model would chose to reformulate these foods rather than lose the HRC – hence, the prevalence of HRCs remains the same as in the baseline scenario but the nutritional quality of foods that carry claims increases to the levels in Models 1a and 2a.

Draft EU nutrient profile model for the regulation of health and nutrition claims

Draft Commission Regulation of 17th March 2009: Specific nutrient profiles and conditions of use, which food or certain categories of food must comply with in order to bear nutrition or health claims (personal communication).

**Food category**		**Thresholds**		
		**Sodium (mg/100g)**	**Saturated fat (g/100g)**	**Total sugars (g/100g)**
Vegetable oils, butter and spreadable fats, as defined in Article 115 and Annex XV of Council Regulation (EC) No 1234/2007		500	30	10
Products of fruits, vegetables, and seeds except oils	Products of fruits and vegetables products, except oils. Minimum 50g of fruit and/or vegetables, except for nectars covered by Directive (EC) No 112/2001.	400	5	15
	Seeds products, except oils. Minimum 50% of nuts per 100g of finished products.	400	10	15
Meat based products. Minimum 50g of meat or 100g of finished products.		800	8	10
Fishery products, crustaceans, and molluscs. Minimum 50g per 100g of finished products.		800	10	10
Dairy products as defined in Council Regulation (EC) No 1234/2007, Annex XII	Dairy products, except cheeses. Minimum 50g of dairy constituents per 100g of finished products, except for drinks based on fermented milks, Minimum 40g per 100g for drinks based on fermented milks	300	2.6	15
	Cheeses. Minimum 50g of dairy constituents per 100g of finished products	900	20	15
Cereal and cereal products	Cereal and cereal products except breakfast cereals and fine bakery wares. Minimum 50g of cereals per 100g of finished products.	400	5	15
	Biscuits and other fine bakery wares. Minimum 30g of cereals per 100g of finished products for fine bakery wares	500	8	25
	Breakfast cereals. Minimum 50g of cereals per 100g of finished products	500	5	25
Ready meals, soups and sandwiches	Soups. Minimum 200g per serving size.	400	5	10
	Ready meals and sandwiches. Minimum 200g per serving size. Minimum 2 of the following for – 30g fruits, vegetables, and/or nuts, 30g cereals, 30g meat, 30g fish, and/or 30g milk.			
Soy based products	Soy based products containing between 3 and 10% soy protein	300	2.6	15
	Soy based products containing more than 10% soy protein	800	8	10
Non-alcoholic beverages, insofar as they do not qualify for one of the above mentioned food categories		300	2	8
Other foods, insofar as they do not qualify for one of the above mentioned food categories		300	2	10

RESULTS

Table 1. Nutrient intake (per day) under each health-related claim (HRC) scenario

Model	Nutrients from foods...	% of total sales	Energy (kcal)	Protein (g)	Carbohydrate (g)	Total sugars (g)	Total fat (g)	Saturated fat (g)	MUFAS (g)	PUFAS (g)	Fibre (g)	Sodium (g)	Cholesterol (mg)	Fruit (g)	Vegetables (g)
Baseline	with HRCs	37%	558.9	21.2	78.4	35.4	19.3	5.5	9.2	4.3	5.7	0.5	75.1	91.9	54.5
	without HRCs	63%	1347.8	44.9	155.5	70.5	60.4	25.5	20.1	9.9	6.8	1.8	149.0	59.3	76.6
	Total	100%	1906.8	66.1	233.8	105.9	79.7	31.0	29.4	14.2	12.6	2.3	224.1	151.2	131.1
1a	with HRCs	27%	299.2	13.6	45.8	20.3	9.5	2.0	5.4	2.5	4.5	0.2	46.1	93.2	55.3
	without HRCs	73%	1589.2	52.8	182.0	83.3	71.9	30.4	24.0	11.7	7.8	2.2	178.0	61.8	79.8
	Total	100%	1888.4	66.5	227.8	103.7	81.4	32.4	29.4	14.2	12.3	2.3	224.1	154.9	135.1
2a	with HRCs	28%	342.5	15.8	48.0	20.1	11.2	2.7	6.0	2.7	4.3	0.2	54.1	83.2	56.1
	without HRCs	72%	1556.3	51.0	180.5	82.5	70.0	29.6	23.3	11.5	7.8	2.1	170.0	64.3	83.1
	Total	100%	1898.8	66.8	228.5	102.6	81.2	32.2	29.4	14.2	12.1	2.3	224.1	147.6	139.3
1b	with HRCs	37%	468.8	20.2	67.3	24.7	16.8	3.3	9.2	4.3	6.4	0.3	75.1	97.4	57.9
	without HRCs	63%	1347.8	44.9	155.5	70.5	60.4	25.5	20.1	9.9	6.8	1.8	149.0	59.3	76.6
	Total	100%	1816.6	65.1	222.7	95.2	77.2	28.7	29.4	14.2	13.2	2.1	224.1	156.7	134.5
2b	with HRCs	37%	498.7	21.0	69.6	24.6	17.6	3.9	9.2	4.3	5.9	0.3	75.1	91.4	61.6
	without HRCs	63%	1347.8	44.9	155.5	70.5	60.4	25.5	20.1	9.9	6.8	1.8	149.0	59.3	76.6
	Total	100%	1846.5	65.9	225.0	95.0	78.0	29.3	29.4	14.2	12.8	2.1	224.1	150.6	138.2

Table 2. Nutrient intake (by food group) under each health-related claim (HRC) scenario, purchase averages g/ml per person, per week (LCF 2014)

Model	Food group	Purchases	Energy (kcal)	Protein (g)	Total fat (g)	Saturated fat (g)	Carbohydrate (g)	Total sugars (g)	Fibre (g)	Sodium (g)	MUFAs (g)	PUFAs (g)	Cholesterol (mg)	Fruit (g)	Vegetables (g)
Baseline	Potatoes, bread, rice, pasta or other starchy carbohydrates	1518.1	421.4	12.8	5.7	1.5	84.8	6.4	5.3	0.55	1.8	1.6	2.6	0.0	0.0
	Composite foods	556.5	152.8	8.0	8.0	2.7	13.1	2.0	0.7	0.33	3.1	1.6	26.0	0.0	0.0
	Foods and drinks high in fat and/or sugar	2840.2	687.6	6.2	37.8	14.7	86.1	59.3	1.8	0.63	14.1	7.2	40.0	0.0	0.0
	Fruit and vegetables	1973.3	102.1	2.7	1.1	0.2	21.9	20.0	3.3	0.04	0.3	0.4	0.3	1058.4	917.8
	Beans, pulses, fish, eggs, meat and other protein	929.2	260.0	22.3	17.1	5.5	4.7	1.6	1.1	0.50	7.3	2.9	124.8	0.0	0.0
	Dairy or dairy alternatives	1835.4	192.5	12.5	9.6	6.1	14.9	14.6	0.0	0.22	2.6	0.4	29.6	0.0	0.0
	Excluded/Miscellaneous	1187.1	90.4	1.8	0.6	0.2	8.4	2.0	0.3	0.08	0.2	0.1	0.8	0.0	0.0
	ALL FOODS	10839.8	1906.8	66.1	79.7	31.0	233.8	105.9	12.6	2.35	29.4	14.2	224.1	1058.4	917.8
1a	Potatoes, bread, rice, pasta or other starchy carbohydrates	1518.1	408.6	13.0	5.0	1.3	83.1	4.5	5.2	0.56	1.8	1.6	2.6	0.0	0.0
	Composite foods	556.5	151.8	7.8	8.0	2.7	12.9	1.9	0.7	0.33	3.1	1.6	26.0	0.0	0.0
	Foods and drinks high in fat and/or sugar	2840.2	690.5	6.3	39.3	15.7	83.4	59.8	1.7	0.61	14.1	7.2	40.0	0.0	0.0
	Fruit and vegetables	1973.3	100.6	2.7	1.0	0.2	22.3	20.3	3.3	0.04	0.3	0.4	0.3	1084.5	946.0
	Beans, pulses, fish, eggs, meat and other protein	929.2	256.6	22.1	17.2	5.7	4.1	1.4	1.0	0.49	7.3	2.9	124.8	0.0	0.0
	Dairy or dairy alternatives	1835.4	198.7	12.9	10.4	6.7	14.5	14.0	0.0	0.23	2.6	0.4	29.6	0.0	0.0
	Excluded/Miscellaneous	1187.1	81.7	1.6	0.5	0.2	7.6	1.8	0.3	0.07	0.2	0.1	0.8	0.0	0.0
	ALL FOODS	10839.8	1888.4	66.5	81.4	32.4	227.8	103.7	12.3	2.33	29.4	14.2	224.1	1084.5	946.0
2a	Bread, rice, potatoes, pasta, etc.	1518.1	409.6	12.8	5.1	1.3	83.3	4.5	4.9	0.5	1.8	1.6	2.6	0.0	0.0
	Composite foods	556.5	151.5	7.9	8.0	2.7	12.9	1.9	0.7	0.3	3.1	1.6	26.0	0.0	0.0

Model	Food group	Purchases	Energy (kcal)	Protein (g)	Total fat (g)	Saturated fat (g)	Carbohydrate (g)	Total sugars (g)	Fibre (g)	Sodium (g)	MUFAs (g)	PUFAs (g)	Cholesterol (mg)	Fruit (g)	Vegetables (g)
	Foods and drinks high in fat and/or sugar	2840.2	694.4	6.3	39.7	15.7	83.8	59.4	1.7	0.6	14.1	7.2	40.0	0.0	0.0
	Fruit and vegetables	1973.3	101.8	2.8	1.0	0.2	21.7	19.5	3.3	0.0	0.3	0.4	0.3	1033.0	974.9
	Meat, fish, eggs, beans, etc.	929.2	252.6	22.4	16.7	5.5	4.0	1.4	1.0	0.5	7.3	2.9	124.8	0.0	0.0
	Milk and dairy foods	1835.4	198.9	12.8	10.2	6.6	14.4	13.9	0.0	0.2	2.6	0.4	29.6	0.0	0.0
	Excluded/Miscellaneous	1187.1	90.0	1.8	0.6	0.2	8.3	2.0	0.3	0.1	0.2	0.1	0.8	0.0	0.0
	ALL FOODS	10839.8	1867.7	67.6	80.6	31.7	220.0	98.4	11.9	2.1	29.4	14.2	224.1	1085.0	1039.5
1b	Potatoes, bread, rice, pasta or other starchy carbohydrates	1518.1	411.3	13.2	4.9	1.2	83.3	4.6	5.9	0.52	1.8	1.6	2.6	0.0	0.0
	Composite foods	556.5	148.5	7.8	7.8	2.6	12.5	1.9	0.7	0.32	3.1	1.6	26.0	0.0	0.0
	Foods and drinks high in fat and/or sugar	2840.2	637.3	5.5	36.7	13.6	78.3	50.9	1.6	0.53	14.1	7.2	40.0	0.0	0.0
	Fruit and vegetables	1973.3	99.8	2.7	1.0	0.2	22.3	20.4	3.3	0.04	0.3	0.4	0.3	1097.0	941.2
	Beans, pulses, fish, eggs, meat and other protein	929.2	261.1	22.0	17.3	5.1	4.5	1.4	1.3	0.43	7.3	2.9	124.8	0.0	0.0
	Dairy or dairy alternatives	1835.4	181.8	12.2	9.1	5.9	14.5	14.3	0.0	0.20	2.6	0.4	29.6	0.0	0.0
	Excluded/Miscellaneous	1187.1	76.8	1.6	0.5	0.1	7.2	1.7	0.3	0.06	0.2	0.1	0.8	0.0	0.0
	ALL FOODS	10839.8	1816.6	65.1	77.2	28.7	222.7	95.2	13.2	2.11	29.4	14.2	224.1	1097.0	941.2
2b	Potatoes, bread, rice, pasta or other starchy carbohydrates	1518.1	413.1	12.9	5.0	1.2	83.8	4.7	5.6	0.50	1.8	1.6	2.6	0.0	0.0
	Composite foods	556.5	150.2	7.9	7.9	2.7	12.7	1.9	0.7	0.32	3.1	1.6	26.0	0.0	0.0
	Foods and drinks high in fat and/or sugar	2840.2	651.8	5.7	37.7	13.9	79.8	51.4	1.6	0.53	14.1	7.2	40.0	0.0	0.0
	Fruit and vegetables	1973.3	100.2	2.7	1.0	0.2	21.7	19.7	3.3	0.04	0.3	0.4	0.3	1054.5	967.6
	Beans, pulses, fish, eggs, meat and other protein	929.2	253.5	22.5	16.5	5.1	4.3	1.4	1.1	0.46	7.3	2.9	124.8	0.0	0.0
	Dairy or dairy alternatives	1835.4	187.3	12.3	9.3	6.0	14.4	14.1	0.0	0.21	2.6	0.4	29.6	0.0	0.0

**Model**	**Food group**	**Purchases**	**Energy (kcal)**	**Protein (g)**	**Total fat (g)**	**Saturated fat (g)**	**Carbohydrate (g)**	**Total sugars (g)**	**Fibre (g)**	**Sodium (g)**	**MUFAs (g)**	**PUFAs (g)**	**Cholesterol (mg)**	**Fruit (g)**	**Vegetables (g)**
	Excluded/Miscellaneous	1187.1	90.3	1.8	0.6	0.2	8.4	1.9	0.3	0.06	0.2	0.1	0.8	0.0	0.0
	ALL FOODS	10839.8	1846.5	65.9	78.0	29.3	225.0	95.0	12.8	2.12	29.4	14.2	224.1	1054.5	967.6
